# Bilateral Ureteral Obstruction in Children after Appendectomy

**DOI:** 10.1155/2015/740795

**Published:** 2015-07-29

**Authors:** M. Grande, G. Lisi, D. Bianchi, P. Bove, R. Miano, A. Esser, F. De Sanctis, A. Neri, S. Grande, M. Villa

**Affiliations:** ^1^Department of Experimental Science and Surgery, Policlinico “Tor Vergata”, Viale Oxford, 00100 Rome, Italy; ^2^Department of Urology, Policlinico “Tor Vergata”, Viale Oxford, 00100 Rome, Italy; ^3^Department of Prevention and Biomedicine, Policlinico “Tor Vergata”, Viale Oxford, 00100 Rome, Italy

## Abstract

Acute renal failure due to bilateral ureteral obstruction is a rare complication after appendectomy in children. We report a case of bilateral ureteric obstruction in a 14-year-old boy nine days after surgery for an acute appendicitis. After saline-filling of the urinary bladder, transabdominal ultrasound demonstrated bilateral hydronephrosis of moderate degree. No abscess was found with CT but presence of millimetric stones on both distal ureters was shown, with bilateral calyceal dilatation. Cystoscopy revealed inflammatory changes in the bladder base. Following introduction of bilateral ureteric stents, there was rapid normalisation of urinary output and serum creatinine.

## 1. Introduction

Appendicitis is still the common cause of emergency abdominal pain and operations in children and adolescents [1].

If an unperforated appendix has been removed, postoperative complications occur in only 5% of patients. However, in gangrenous or perforated appendices, occurring in more than one-third of all patients with appendicitis, morbidity rates may be 40% [2].

Well known complications include wound infection, perforation, peritonitis, abscess formation, or mechanical bowel obstruction. Urologic complications are uncommon and usually result from right-sided ureteral obstruction, in most cases due to an appendiceal abscess [3].

Bilateral ureteric obstruction is a rare complication. We report a case of abdominal pain and then anuria due to bilateral ureteral obstruction, not related to an abscess, in a 14-year-old boy, several days after appendectomy for acute appendicitis.

## 2. Case Presentation

In 2013, a 14-year-old boy was admitted to our department (Department of General Surgery) with abdominal pain localized in the right lower abdominal quadrant, with no fever and no vomiting. He had no previous history of surgery or other diseases. Clinical examination showed the presence of peritonitis in the right iliac fossa (McBurney's sign was positive), with fever (38°C). Laboratory values were white blood cell count 14.090 *μ*L (range 4.300–10.800) (neutrophilia 82.5%), creatinine 0.70 mg/dL (range 0.50–1.20), and azotemia 29 mg/dL (range 15–50). Following the worsening of the clinical condition, the young patient underwent surgery for laparoscopic appendectomy. An acute appendicitis with consensual serositis and haemorrhagic necrosis was reported.

**Figure 1:**
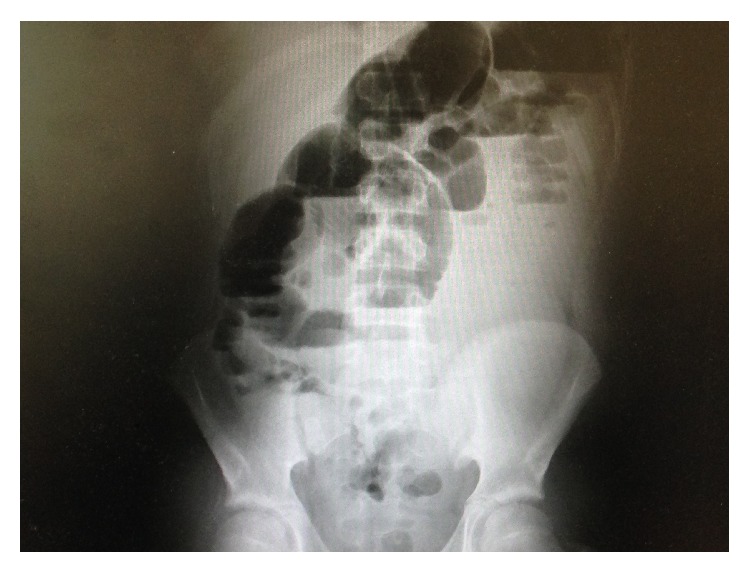
Small bowel loop.

He received antibiotics preoperatively and postoperatively (ceftazidime 250 mg and metronidazole 500 mg, both three times daily). The early postoperative course was uneventful, with normal urinary output (1,600 mL/d) and white blood cell count 8.500 *μ*L (neutrophilia 80.5%). Two days after surgery the patient complained of nausea with vomiting and following right-sided flank pain. He was afebrile. An abdominal CT scan without contrast-enhanced imaging was performed; it showed “marked suffusion of loose tissue in the side of the previous appendectomy likely due to abscess with pneumatosis widespread and distension of both the small bowel and the colon with multiple air-fluid levels.” The day after Rx abdomen showed dilated small bowel loops out of proportion to gas in the colon (Figure 1).

**Figure 2:**
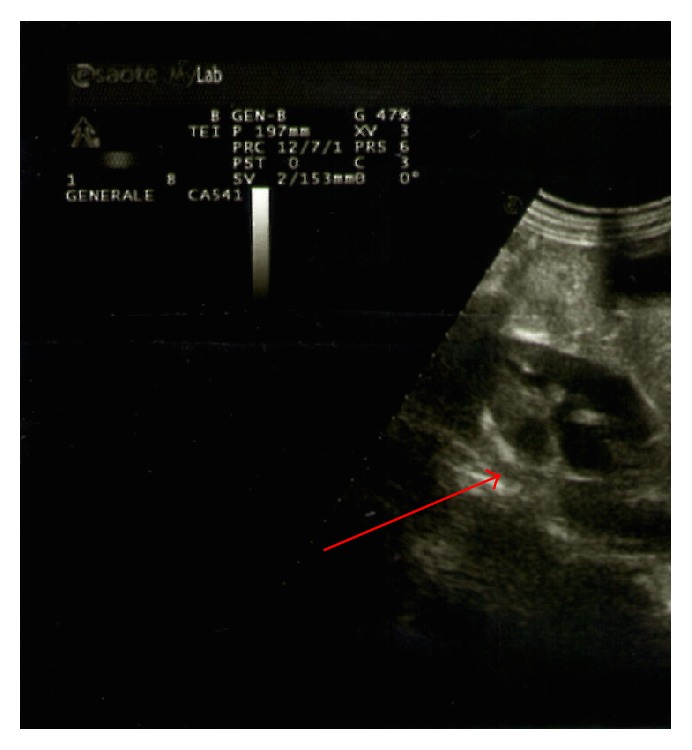
Right hydronephrosis.

**Figure 3:**
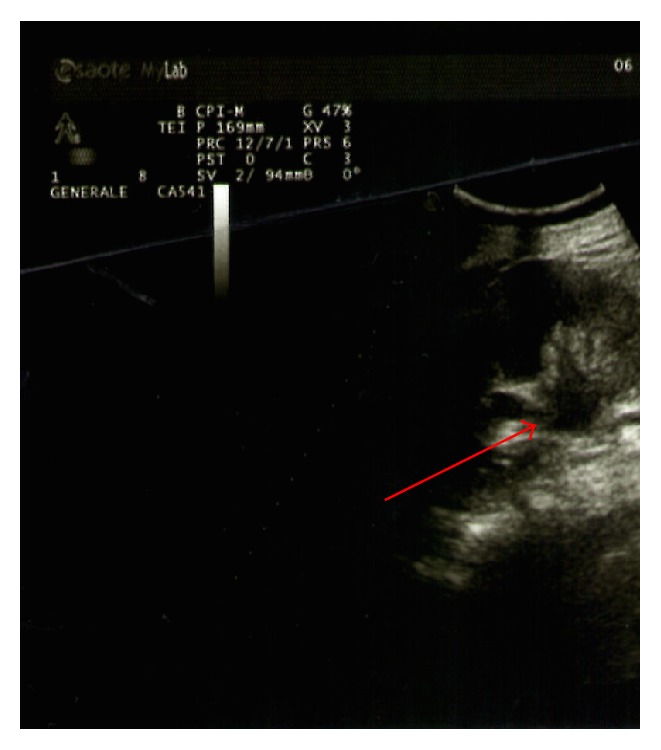
Left hydronephrosis.

Blood tests were normal. The patient underwent exploratory laparoscopy which permitted excluding the presence of an abscess in the right iliac fossa, showing only the presence of small bowel adhesions with the wall. Therefore, adhesiolysis was performed with placement of tubular suction drainage. The postoperative course was regular until the fourth postoperative day after the second operation, when diffuse abdominal pain with vomiting, no signs of peritoneal reaction, absent peristalsis, and no fever reappeared. A new CT study was performed. There were no alterations of abdominal parenchyma. At the site of the previous surgery appreciates widespread inhomogeneities perivisceral loose tissue in the presence of minimal fluid component. Minimum air-fluid levels are appreciable in the small bowel. The day after ultrasound of the urinary tract demonstrated bilateral hydronephrosis of moderate degree (Figures 2 and 3).

**Figure 4:**
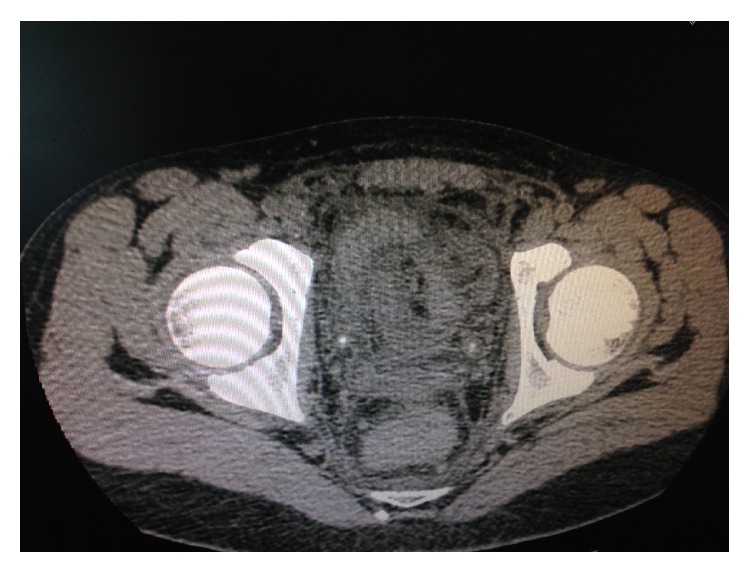
Stones on both distal ureters.

White blood cell count was 20.020 *μ*L (neutrophilia 88.1%), C-reactive protein was 18.69 mg/L (range 0–3), and serum creatinine was 2.10 mg/dL. In the next morning, urinary output decreased to 250 mL/d, and urinalysis showed more than 200 red blood cells per high-power field, pH 6.14. Blood test results were as follows: Hb 12.50 g/dL, hematocrit 35.80%, white blood cell 19.740, glucose 108 mg/dL, blood urea 6.8 mg/dL, creatinine 4.50 mg/dL, azotemia 51 mg/dL, and C-reactive protein (CRP) 26.89. Upon clinical suspicion of ureter obstruction, the patient underwent CT scan without contrast-enhanced imaging. This examination excluded an abscess but showed the presence of millimetric 280-Hounsfield Unit stones on both distal ureters, left renal pelvis, and calyces and in the urinary bladder. Besides, CT revealed bilateral calyceal dilatation (Figure 4).

Cystoscopy under general anaesthesia showed inflammatory changes in the bladder base and a swollen right-sided ostial region with a whitish “plug” protruding from the orifice. Urine sample was collected to perform a bacteriologic examination, which showed no bacterial growth.

Then a 6-French semirigid ureteroscope was introduced, confirming the presence of multiple millimetric soft stones that were removed by an endoscopic basket. A right pyelogram was performed along with positioning of 6-French diameter, 24 cm length double-J stent on the right ureter. The same procedure was performed on the left ureter with prompt stones drainage as the guide wire was introduced. A ureteral stricture (probably reactive, with edematous ureteral walls) was revealed on the distal ureter, about 2-cm before the ureteral orifice. We decided to stop the ureteroscopic procedure. A left pyelogram was performed and a 5-French, 24-cm length ureteral stent was positioned along the left ureter. On the following days, the patient had normal urine production. Flank pain and nausea subsided, and serum creatinine decreased to 1.00 mg/dL. He was discharged three days after cystoscopy. The ureteral stents were removed 1 month after the procedure.

## 3. Discussion

We presented an unusual case of bilateral ureteral obstruction after laparoscopic appendectomy in children. Acute urinary retention, as a complication of appendicitis, was first described by Dever et al. [4].

The last report found on PubMed, with a review of the literature, dates back to 2005 [5] when the authors described a case of anuria in an 11-year-old boy, 5 days after surgery for a perforated appendix. Acute postrenal failure is rare in childhood. It can be caused by congenital urinary tract malformations, immunorheumatologic causes, ureteral localization of infections, neoplastic intrinsic ureteral obstructions, extrinsic ureteral obstructions, and iatrogenic trigonal obstruction or inflammation [6]. Bilateral obstruction is less common but may also be due to mechanical obstruction by an abscess. However, without abscess formation as in our case report, bilateral obstruction is a rare complication [5].

Ureteral oedema has been postulated as possibly triggered by a localized peritoneal reaction to intraoperative bacterial contamination, with boys more susceptible because their appendix is located closer to the bladder, while in girls internal genitalia are situated between the appendix and the bladder [7]. Inflammatory changes of the posterior bladder wall are described as a complication to severe appendicitis (gangrenous or perforated) and probably are related to a localised peritonitis [8, 9]. The subsequent oedema may compromise the urinary flow and in some cases give rise to complete ureteral obstruction [5].

There are today several imaging modalities used for the investigation of urinary tract obstruction in children. Magnetic resonance imaging and renography are less practical tools in the acute setting. Intravenous pyelography includes the use of a contrast agent with possible nephrotoxic effect, and the display of pathology in the distal parts of the ureters is in many cases suboptimal. Computer tomography (CT) without contrast agents has been introduced as a rapid method to image ureterolithiasis in adults. However, the considerable radiation dose of a CT of the abdomen should give rise to caution [9]. The ultrasound diagnosis of ureteral obstruction is characterised by detection of hydronephrosis, detection of ureteric jets from the orifices with color Doppler, and examination of the ureter [10].

In fact, in our case, ultrasound study after two CT showed an initial bilateral hydronephrosis. In our patient, not having found an evident cause, such as formation of an abscess, it would be useful to proceed with a metabolic study, but the patient was lost to followup.

## 4. Conclusion

In conclusion, it is difficult to make a diagnosis before the onset oliguria in a young patient with abdominal pain after appendectomy. The surgeon as well as the radiologist or any other consultants are led to think that the cause of the symptoms reported by the patient is to be charged to results of previous appendectomy. When flank pain appears, nausea and oliguria should lead to suspicion, even if no hydronephrosis is detected.

Early diagnosis and intervention are important to prevent irreversible renal damage, and ureteric stenting is the treatment of choice. In our patient, this procedure had an immediate clinical effect with complete remission of pain, ready bowel recanalization, and normalisation of diuresis and serum creatinine.

Ureteral obstruction secondary to appendectomy could be related to dehydration, although in our opinion further studies would be necessary to highlight critical points and to evaluate metabolic aspects.
